# Naphey’s Therapeutics

**Published:** 1892-09

**Authors:** 


NAPHEY'S THERAPEUTICS. In two handsome volumes of 1000 pages each. Vol. I—General Medicine and Diseases of Children; Vol. II—General Surgery, Obstetrics and Diseases of Women. Ninth Edition. Revised and enlarged. Edited by Allen J. Smith, M. D., and J. Aubrey Davis, M. D. Royal octavo. Half Russia, marbled edges, $6 per volume net. Each volume sold separately. Subscription edition; sold only by subscription.

The JOURNAL is in receipt of Vol. 1 of this standard work. Naphey's Therapeutics is too well known to the medical profession to require any commendation at our hands; it has been a popular favorite for many years; but like all other standard works, the advances and discoveries in medicine having made many and radical changes in the treatment of disease, a revision was necessary to make the work applicable to the practice of modern medicine. The enterprising publishers recognizing that there was a demand for such revision have brought this out—the ninth edition—in fine style, mechanically, and with all that has been abandoned, eliminated, and all that has been acquired in the knowledge of the action of medicines and the latest achievements of therapeutic science added; and it now represents the therapeutics of to-day. "The precise formulæ, specific directions and methods of treatment recommended by the most eminent American and foreign practitioners are given; and the full resources of the materia medica are grouped under the various diseases to which they are applicable." All the new remedies are brought forward and the modes of employment and their relative value are set forth. In brief the work is an excellent "practice of medicine;" it is a library within itself. It will be seen that Diseases of Women and Children have been included, instead of being published separately as before.

